# A novel chaos-based approach for constructing lightweight S-Boxes

**DOI:** 10.1038/s41598-025-20019-4

**Published:** 2025-09-30

**Authors:** Sohila H. Tolpa, Mohamed A. Abdelhamed, El-Sayed Soliman A. Said, Mohamed Yasin I. Afi

**Affiliations:** 1 Department of Communications and Computers Engineering, Higher Institute of Engineering, El Shorouk Academy, El Shorouk City, 11837 Egypt; 2 Department of Electrical Engineering, Faculty of Engineering, Al-Azhar University, Nasr City, 11884 Egypt

**Keywords:** Electrical and electronic engineering, Mathematics and computing

## Abstract

Cryptography is the science of using specific secret writing techniques to convert an original message into a coded form for safe transmission over public networks. While conventional cryptographic techniques are efficient on resource-rich devices (e.g., PCs, servers, smartphones), they may perform poorly on the Internet of Things (IoT) devices with limited resources (e.g., Radio Frequency Identification (RFID) tags, sensors). Consequently, a specialized approach known as Light-Weight Cryptography (LWC) is necessary. The Substitution-Box (S-Box) is a crucial and distinct component in constructing cryptographic algorithms that introduces nonlinearity between inputs and outputs. This paper uses chaotic maps to present a novel approach for constructing 4 × 4 S-Boxes tailored for LWC with strong cryptographic properties. The method initially employs an enhanced sine map, followed by a combination of an enhanced logistic map and an enhanced tent map. This approach optimizes multiple parameters by a defined security threshold. The evaluation of the generated 4 × 4 S-Boxes confirms their optimal performance in the context of the Strict Avalanche Criterion (SAC), the Bit Independence Criterion (BIC), and enhanced resistance to Side Channel Attack (SCA), outperforming existing S-Boxes. Furthermore, successfully obscuring image features demonstrated their effectiveness in image encryption. We assessed the hardware efficiency of one of the generated S-Boxes by calculating its Gate Equivalent (GE) using the NanGate 45nm technology. The highly secure S-Boxes constructed in this study can serve as replacements for similar-sized S-Boxes in existing algorithms or be utilized to construct lightweight block cipher algorithms.

## Introduction

Cryptography is a field of specialized secret writing techniques which change the original message into a coded message with an unreadable format for its transmission over public networks in the presence of adversaries^1^. For this purpose, we need a system or procedure for converting data or messages into secret codes. Such systems are known as Cryptosystems. Thus, a good crypto system should meet confidentiality, integrity, non-repudiation and authentication criteria^2^. A typical cryptosystem has five major components: Plaintext, Cipher text, Encryption Algorithm, Decryption Algorithm and Key. When considering the design aspects of a cryptosystem, any cryptography algorithm can be divided into two categories: symmetric key and asymmetric key cryptography. A symmetric key cryptosystem is further classified into either a stream cipher or a block cipher^3^. The block ciphers are designed based on Shannon’s theory of confusion and diffusion; Claude Shannon is considered the first person to introduce the two primitive cryptographic operations (Substitution and Permutation) in 1949^4^. The Substitution-Box (S-Box), a key nonlinear component of block ciphers, is critical to assuring the security of the encryption process^5^ since it offers confusion and diffusion.

The design of the S-Boxes plays a crucial role in determining the efficiency of a block cipher. Several techniques to construct S-Boxes have been proposed, including the utilization of Gaussian Distribution^5^, Linear Fraction Transformation^6^, Improved Sine Cosine Algorithm^7^, Fuzzy Logic^8^, Cyclic Group Curves^9^, or Genetic Algorithm^10^. Another approach gaining attention is the application of Chaos Theory, which focuses on systems that exhibit extreme sensitivity to initial conditions. Chaotic systems show unpredictable behaviors, making them ideal for secure communication and encryption. In chaotic cryptography, chaotic systems perform encryption operations such as substitution and permutation. These systems ensure that even slight variations in input will result in completely different outputs. Chaotic maps have been widely explored for the construction of S-Boxes. To create an S-Box, it is necessary first to identify an appropriate chaotic system and then develop an efficient approach. Chaotic systems are categorized into two types, one-dimensional and multi-dimensional, each having distinct advantages for cryptographic applications^11^. Research on constructing S-Boxes using chaotic systems is discussed in^12–17^. Additionally, some studies integrate chaotic systems with other approaches to create S-Boxes as discussed in^18–20^.

The referenced research findings are less applicable when dealing with smaller S-Boxes. Due to their large size (8 × 8), these S-Boxes are not suitable for resource-constrained Internet of Things (IoT) devices (e.g., sensors, Radio Frequency Identification (RFID) tags, and actuators), their limited memory, small physical area to implement, low computational power, and low energy have resulted in higher security requirements. Its lighter version, Light-Weight Cryptography, could address such resource limitation challenges. The design of lightweight cryptographic algorithms often involves using smaller, more efficient S-Boxes. For instance, the 3-bit S-Box^21^ is both hardware and software-efficient due to its cost-effective implementation on extremely low-cost RFID tags. At the same time, it is prone to attacks due to the limited variety of possibilities for constructing different S-Boxes. While 8-bit S-Boxes are comparatively more secure than the 3-bit S-Box, they are more expensive regarding resource utilization. A common approach in lightweight cipher design is to utilize 4-bit S-Boxes. These S-Boxes are small enough to be implemented efficiently while still providing an adequate level of security. The reduced size of 4-bit S-Boxes helps minimize the hardware footprint and computational overhead, making them suitable for IoT applications.

The 4×4 S-Box is used by popular lightweight cryptographic algorithms such as PRESENT^22^ and has been standardized by the International Organization for Standardization/International Electrotechnical Commission. (ISO/IEC). PHOTON-Beetle^23^, EPCBC (Electronic Product Code Block Cipher)^24^ and LED^25^ use the same S-Box as PRESENT algorithm. The PHOTON-Beetle^23^ is another lightweight cryptographic algorithm standardized by the National Institute of Standards and Technology (NIST). Apart from the previously mentioned renowned S-Boxes of PRESENT and PHOTON-Beetle, 4 × 4 S-Boxes are employed in the algorithms that made it to the second round of NIST’s lightweight algorithm selection. These algorithms include ELEPHANT^26^, GIFT^27^, KNOT^28^, PYJA MASK^29^, and SPOOK^30^. Among these, PHOTON-Beetle, ELEPHANT, and GIFT successfully advanced to the finals of the NIST competition. There are other widely recognized algorithms, such as PRINCE^31^, KLEIN^32^, PRIDE^33^ and RECTANGLE^34^. Recent studies, as referenced in^35–37^, further explore the applications and designs of 4 × 4 S-Boxes.

Evaluating the S-Boxes in previous algorithms or any other S-Box involves several essential criteria. Some of the most important are the balanced, bijective property, Non-linearity (NL), Strict Avalanche Criterion (SAC), Bit Independence Criterion (BIC), Algebraic Degree, Fixed and Opposite Fixed Points (FP and OFP), Differential Approximation Probability (DAP), and Linear Approximation Probability (LAP).

The rest of the paper is organized as follows: Section "Related works" discusses a few recent related works. Section "Background" introduces the characteristics of cryptographically resilient S-Boxes and 1-D chaotic maps. Section "Proposed S-boxes construction methodology" describes the proposed approach for constructing S-Boxes and provides detailed insights into the cryptanalysis findings. Section "Application and hardware efficiency" provides thorough insights into the image encryption application of the S-Boxes and the hardware implementation. The last section concludes the paper.

## Related works

Many studies have utilized chaotic maps in cryptography, particularly in designing and developing S-Boxes. In this section, we highlight recent research that is specifically focused on 4 × 4 S-Boxes. In Ref.^37^, the authors used an Enhanced Tent Map to construct a robust 4 × 4 S-Box (ET-S-Box), achieving optimal SAC and BIC-SAC values of 0.5. However, Side-Channel Attack (SCA) resistance parameters are insufficient, making it prone to side-channel attacks.

In Ref.^17^, the authors used the logistic map to initialize the jellyfish’s initial positions. Then, they employed a modified version of the Artificial Jellyfish Search (JS) algorithm to construct a robust 4 × 4 S-Box (JS-S-Box). They evaluated the basic security properties of this S-Box but did not assess its resistance to Side Channel Attacks (SCA). In ref.^1^, the authors used an Enhanced Logistic to construct a 5 × 5 S-Box designed for lightweight devices. The algorithm employed iteratively computes 32 values multiplied by 256 to expand the domain and then takes these values modulo 32 to yield 32 values of the S-Box. The NL of the S-Box was calculated as 2.625 using hamming distance (not the Walsh-Hadamard matrix, since 5 is not a multiple of 2). The LP and DP are 0.25, while SAC and BIC-SAC fall short of the optimal value of 0.5. The authors did not analyze SCA.

The latest comprehensive study consolidates research findings on generating 8×8 S-Boxes using various methods, including chaos-based approaches (logistic map, square map, sin map, cosine map, tent map and circle map), and is presented in^38^.

In Ref.^39^, Asim et al. introduced a novel approach for constructing strong bijective S-Boxes using a piecewise-linear chaotic map. This method partitions the input and output spaces of the S-Box and applies the chaotic map to each partition to perform byte substitutions. The results show an average BIC of 108 for the proposed S-Box, which is higher than that of the others. However, the NL and SAC values are not optimal. Additionally, the latest comprehensive study on various methods for generating 8×8 S-Boxes, including chaos-based approaches. Alhadawi et al.^40^ introduced a novel algorithm for designing S-Boxes by employing discrete chaotic maps in combination with the cuckoo search algorithm. The technique generates candidate S-Boxes using chaotic maps and evaluates them based on a fitness function that incorporates various cryptographic criteria. The results show that the proposed S-Box has a better average NL of 108.5 and LP of 0.1094 compared to others, although the SAC and BIC-SAC values are not optimal.

The 4 × 4 S-Boxes that are used in most popular lightweight cryptographic algorithms have been analyzed also such as PRESENT^22^, ELEPHANT^26^, GIFT^27^, KNOT^28^, PYJAMASK^29^, SPOOK^30^, PRINCE^31^, KLEIN^32^, PRIDE^33^, RECTANGLE^34^ and FEATHER^41^. After analyzing these S-Boxes, we have concluded that they do not meet the optimal criteria regarding specific characteristics related to side-channel attack (SCA) resistance and BIC and SAC.

In^42^, the authors proposed a novel method for creating S-Boxes by combining the Grey Wolf Optimizer (GWO) with a discrete chaotic map. The aim is to optimize S-Boxes’ properties, which are crucial for cryptographic applications. The authors aim to enhance the nonlinearity of S-Boxes, achieving an average of 109 using XGWO.

After reviewing the literature, the following points can be concluded.

- The method of leveraging chaotic maps has been widely employed in the S-Box generation in recent years^1,12,37^. However, published S-Boxes using chaos for lightweight cryptography are limited, and recent results have yet to optimize many criteria.
- The simplicity of computation and implementation in one-dimensional chaotic maps makes them attractive for S-Box generation algorithms. However, improving their chaotic behavior and addressing their limited chaotic range are crucial steps to enhance their effectiveness^11,18^ to improve them for S-Boxes generation approaches.
- Published research generally aims to create S-Boxes based on specific criteria, such as nonlinearity or BIC, rather than optimizing multiple criteria simultaneously.
- Most of the published research on S-Box generation does not consider the study of parameters that enhance the S-Box’s resistance to side-channel attacks (SCA)^43,44^.

The main objective of this research is to construct lightweight S-Boxes with optimal SAC and BIC parameters. Constructing S-Boxes with inherent resistance to side-channel attacks remains a significant challenge in cryptography. Building new parameters proposed in recent studies^43–45^ that enhance the resistance of S-Boxes against SCA, this study aims to optimize these parameters during the S-Box design process. This article employs chaotic maps in the creation of S-Boxes.

The key contributions of this work are summarized as follows.

- This study introduces a method for constructing 4 × 4 S-Boxes, initially using the “Enhanced Sin Map” and then combining the “Enhanced Logistic” and “Enhanced Tent.” The resulting combined system (ELET) demonstrates chaotic behavior over a broad range of parameters, making it suitable for implementing the proposed approach.
- A novel approach is proposed for constructing lightweight S-Boxes based on multiple security criteria. The main objective of the approach is to optimize SAC and BIC criteria, along with parameters related to resistance against SCA. Adjusting these parameters allows the approach to optimize any criteria and is suitable for S-Boxes of various sizes.
- A 4 × 4 S-Box was constructed using the two aforementioned systems. We have thoroughly analyzed and evaluated all key aspects of the S-Boxes to ensure their security. The cryptanalysis results demonstrate the exceptional performance of the constructed S-Boxes in terms of SAC, BIC, and SCA criteria. The average SAC and BIC-SAC values for the S-Boxes reach 0.5, the optimal value. The parameters related to resistance against side-channel attacks outperform those of most other S-Boxes. At the same time, the remaining criteria are comparable to those of other S-Boxes.
- The effectiveness of the proposed S-Boxes in obscuring image features is demonstrated through image encryption applications. One of the proposed S-Boxes, the ELET S-Box, was selected for detailed hardware analysis, where its gate requirements (e.g., AND gates, OR gates, etc.) were computed. Its Gate Equivalent (GE) was determined using NanGate 45nm technology, and its power consumption was evaluated, confirming its suitability for lightweight cryptographic applications.

## Background

The security properties of a strong S-Box and an overview of one-dimensional chaotic maps are covered in this section.

### Cryptographic properties of a strong S-box

Substitution boxes play a vital role in cryptography. This subsection outlines the methodology for evaluating key criteria in designing strong S-Boxes, including balancedness, bijectivity, NL, SAC, BIC, Algebraic Degree, DAP, LAP, FP and OFP^46^. Additionally, it covers S-Box parameters that help protect against DPA (Differential Power Analysis).

- Balancedness: Each Boolean function *f*: GF(2^*n*^)$\to$ GF(2) of the S-Box should exhibit balancedness, meaning the number of zeros and ones in its truth table should be equal.
- Bijectivity: For a given Boolean function *f*: GF(2^*n*^)$\to$ GF(2) is considered bijective if and only if all linear combinations of columns are balanced. The bijective property is satisfied if for the Boolean functions *f*_i_ (for 1 ≤ *i* ≤ n) of the S-Box:
  1 $${H}_{wt}\left(\sum_{i=1}^{n}{c}_{i}{f}_{i}\right)= {2}^{n-1}$$
  where *c*_*i*_ ϵ {0,1}, and *H*_*wt*_ is the Hamming weight^37^.
- Nonlinearity: An *n*-bit S-Box can be viewed as a collection of *n* Boolean functions defining the mapping between the input and output (*S* = (*f*_1_,* f*_2_, …, *f*_*n*_)). Consequently, the S-Box’s nonlinearity is dictated by the nonlinearity of its component Boolean functions. The Non-linearity, NL(*f*), of any Boolean function *f* is measured by evaluating its deviation from affine functions^47^, typically using Walsh transform or Walsh Hadamard matrices. A higher level of nonlinearity indicates that the S-Box is immune to linear cryptanalysis. Enhancing its security against such attacks. For an S-Box S: GF(2^*n*^)$\to$ GF (2^*n*^), where $S\left(u\right)$ = $v$ for $v\in$ GF(2^*n*^) and $u\in$ GF(2^*n*^). The nonlinearity can be calculated as Eq. (2)^48^.
  2 $${NL}_{f}={2}^{n-1}-\frac{1}{2}\text{max}\left|W\left(u,v\right)\right|$$
  where $W\left(u,v\right)$ is Walsh transform, defined as:
  3 $$W\left(u,v\right)= \sum_{x\in GF\left({2}^{n}\right)}{\left(-1\right)}^{v.f\left(x\right)\oplus u.x}$$
- Strict Avalanche Criterion: The avalanche effect was first proposed by Feistel, H. Later, Kam and Davida introduced the concept of completeness, and finally, Webster and Tavares developed the Strict Avalanche Criterion (SAC) by combining the ideas of avalanche and completeness in S-Boxes. SAC specifies that flipping an individual input bit should result in each output bit flipping with a probability of one-half. The Strict Avalanche Criterion (SAC) is a critical quality metric for evaluating S-Boxes. For robust cryptographic security, the SAC value of an S-Box should ideally be as close as possible to 0.5, indicating that flipping a single input bit produces output changes with optimal randomness and diffusion. The independence matrix values of an *n*-bit S-Box are defined in Eq. (4). The SAC value of an S-Box is taken as the average of the values of ${p}_{i,j}$^37^.
  4 $${p}_{i,j}=\frac{1}{{2}^{n}}\sum_{x\in {{\varvec{F}}}_{2}^{n}}{f}_{i}\left(x\right)\oplus{f}_{i}\left(x \oplus{d}_{j}\right)$$
- Bit Independence Criterion: The Bit Independence Criterion (BIC), another essential property introduced by Webster and Tavares^49^, ensures that flipping any specific input bit results in an independent and unpredictable change in all output bits. Specifically, a change in the *i*th input bit should produce independent changes in the *j* and *k* output *i*, *j*, *k* ∈ {1, 2, …, *n*} and *j* ≠ *k*. For an S-Box, consider two output bits, *f* (*r*) and *f* (*s*), where *r* $\ne$ *s*, the S-Box satisfies the BIC if *f* (*r*) $\oplus$ *f* (*s*), is a highly nonlinear function and comes closer to satisfying the SAC. Thus, the Bit Independence Criterion (BIC) can be evaluated by analyzing two key aspects: the nonlinearity (BIC-NL) and the Strict Avalanche Criterion (BIC-SAC) functions created by performing XOR operations on the output functions. Therefore, the assessment of BIC involves combining the calculations of nonlinearity and SAC.
- Algebraic Degree: The Algebraic Degree of a Boolean function *f :* GF(2^*n*^) → GF (2) is defined as the degree of the highest-order term in its Algebraic Normal Form (ANF) with a non-zero coefficient. A higher algebraic degree is generally preferred for cryptographic strength, contributing to better resistance against algebraic attacks^50^.
- Differential Approximation probability: The differential uniformity of an S-Box is a critical property for its effectiveness in cryptography. This characteristic is quantified using the Differential Approximation Probability (DAP), which assesses how changes in the input of the S-Box, whether sequence or value, affect the output^51^. The degree of differential transformation substantially impacts the security and resistance of an S-Box in cryptographic applications. A lower DAP is preferred, implying a reduced correlation between input and output differences. Differential cryptanalysis^52^, a statistical attack method, exploits the characteristics of an S-Box’s Differential Distribution Table (DDT). Reducing the DAP value strengthens the S-Box, enhancing its resistance to such attacks. The DAP is defined as Eq. (5)^41^.
  5 $$DAP\left(\Delta x\to \Delta y\right)= \frac{\#\{x \in \text{\rm X} \left| S\left(x\right) \right.\oplus S\left(x\oplus\Delta x\right)= \Delta y\}}{{2}^{n}}$$
  where $\text{\rm X}$ represents a list of all potential input values and $n$ is the number of input bits. The term DAP indicates the maximum likelihood of observing a specific output difference $\Delta y$ corresponding to a given input difference $\Delta x$.
- Linear Approximation Probability: Linear Approximation Probability LAP is a metric used in linear cryptanalysis to measure the strength of an S-Box or a cryptographic system against linear attacks. It is defined by Eq. (6)^37^. LAP quantifies the likelihood that a specific linear relationship between an S-Box’s input and output bits holds true. A higher LAP indicates a stronger, highlighting a potential linear equation related to the input and output. Conversely, the security of an S-Box improves as its LAP decreases, with lower values indicating greater resistance to such attacks.
  6 $$LAP= {\text{max}}_{\Delta x,\Delta y\ne 0}\left\{\frac{\#\left\{x \in \text{\rm X} \right| x . \Delta x = S\left(x\right) . \Delta y\}}{{2}^{n}}- \frac{1}{2}\right\}$$
  here, $x$ represents the set of all possible inputs, with a cardinality of 2^*n*^, and $\Delta x$ and $\Delta y$ denote the input and output differentials, respectively.
- Fixed and Opposite Fixed Points: Fixed Points (FP) and Opposite Fixed Points (OFP) are essential properties of an S-Box in cryptography. Consider an S-Box $S$: GF(2^*n*^)$\to$ GF (2^*n*^) and for *u* $\in$ GF(2^*n*^), A fixed point FP of an S-Box occurs when $S$ (*u*) = *u* and an opposite fixed point occurs when $S$ (*u*) = $u{\prime}$. An S-Box that lacks fixed points and opposite fixed points is generally regarded as more resistant to differential cryptanalysis than one with such points.
- Side Channel Analysis: Evaluating the side-channel resistance of lightweight ciphers is essential for ensuring their security in practical implementations, particularly in resource-constrained environments. A key component that influences a cipher’s vulnerability to Side-Channel Attacks (SCAs) is the S-Box, as it plays a significant role in both the nonlinearity and the overall cryptographic strength^43^. Various metrics have been proposed to assess the inherent resistance of S-Boxes to SCAs. These include the Signal-to-Noise Ratio (SNR)^44,53^ in Differential Power Analysis (DPA), where a smaller SNR(S) indicates better resistance of S against DPA, Transparency Order^45,54,55^, and the Confusion Coefficient^56^. A lower Minimum Confusion Coefficient typically correlates with higher resistance to SCAs, while a lower Transparency Order can indicate stronger protection against Differential Power Analysis (DPA). Transparency orders and confusion coefficients are the most commonly used metrics for comparing and selecting optimal S-Boxes with strong SCA resistance^43^. The original Transparency Order (TO)^54^ and the Modified Transparency Order (MTO)^55^ have been widely used for selecting 4 × 4, 6 × 6, and 8 × 8 S-Boxes^57^. However, it has been noted that both TO and MTO have flaws. The Revisited Transparency Order (VTO) concept was introduced to address these issues in^45^. The order of the most importance is VTO, followed by MTO and TO. The calculation method for determining the values of TO, MTO, and VTO is presented in Eqs. (7), (8), and (9), respectively^45^.

7 $$\begin{aligned} TO\left( S \right) & = \max _{{\beta \in F_{2}^{m} }} ~(\left| {m - 2Hwt\left( \beta \right)} \right| \\ & - \frac{1}{{2^{{2n}} - 2^{n} }}~\mathop \sum \limits_{{a \in F_{2}^{{n^{*} }} }} \left| {\mathop \sum \limits_{{i = 1}}^{m} \left( {\left( { - 1} \right)^{{\beta _{i} }} \mathop \sum \limits_{{x \in F_{2}^{n} }} \left( { - 1} \right)^{{f_{i} \left( x \right) \oplus f_{i} \left( {x \oplus a} \right)}} } \right)} \right|) \\ \end{aligned}$$

8 $$\begin{gathered} ~MTO\left( S \right) = \max _{{\beta \in F_{2}^{m} }} ~(m - \frac{1}{{2^{{2n}} - 2^{n} }} \hfill \\ \mathop \sum \limits_{{a \in F_{2}^{{n^{*} }} }} \mathop \sum \limits_{{j = 1}}^{m} |\mathop \sum \limits_{{i = 1}}^{m} \left( {\left( { - 1} \right)^{{\beta _{i} + \beta _{j} }} \mathop \sum \limits_{{x \in F_{2}^{n} }} \left( { - 1} \right)^{{f_{i} \left( x \right) \oplus f_{j} \left( {x \oplus a} \right)}} } \right)|) \hfill \\ \end{gathered}$$

9 $$\begin{gathered} VTO\left( S \right) = \max _{{\beta \in F_{2}^{m} }} ~(m - \frac{1}{{2^{{2n}} - 2^{n} }} \hfill \\ \mathop \sum \limits_{{a \in F_{2}^{{n^{*} }} }} \left| {\mathop \sum \limits_{{j = 1}}^{m} \mathop \sum \limits_{{i = 1}}^{m} \left( {\left( { - 1} \right)^{{\beta _{i} + \beta _{j} }} \mathop \sum \limits_{{x \in F_{2}^{n} }} \left( { - 1} \right)^{{f_{i} \left( x \right) \oplus f_{j} \left( {x \oplus a} \right)}} } \right)} \right|) \hfill \\ \end{gathered}$$

Equation (10) determines the calculation of the minimum confusion coefficient, as described in^58^.

10 $$\begin{aligned} \left( {k^{*} ,k} \right) & = ~E\left\{ {\left( {\frac{{Y\left( {k^{*} } \right) - Y\left( k \right)}}{2}} \right)^{2} } \right\} \\ & = ~E\left\{ {\left( {\frac{{Hwt\left( {S\left[ {x \oplus k^{*} } \right]} \right)~ - ~Hwt\left( {S\left[ {x \oplus k} \right]} \right)}}{2}} \right)^{2} } \right\} \\ \end{aligned}$$

where $Y\left({k}^{*}\right),Y\left(k\right)$ are the predicated intermediate values depending on ${(k}^{*},k),$ $x$ is the plaintext, $k$ denotes the secret key and ${k}^{*}$ denotes a hypothesis key and *E* is the expectation value. The Eq. (11) for calculating SNR is described in^44^.

11 $$\begin{aligned} SNR\left( S \right) & = ~\frac{{m~.~~2^{n} }}{{\sqrt {\mathop \sum \nolimits_{{a \in F_{2}^{n} }} [\mathop \sum \nolimits_{{i = 1}}^{m} w\left( {f_{i} \oplus \varphi _{\alpha } } \right)]^{4} } }} \\ & = ~~\frac{{m~.~~2^{n} }}{{\sqrt {\mathop \sum \nolimits_{{a \in F_{2}^{n} }} [\mathop \sum \nolimits_{{i = 1}}^{m} \mathop \sum \nolimits_{{x \in F_{2}^{n} }} ( - 1)^{{f\left( x \right) \oplus \alpha .x}} ]^{4} } }}~~ \\ \end{aligned}$$

The *m* and* n* parameters in all previous equations represent the dimension of an S-Box; the symbol *f* denotes a Boolean function associated with the S-Box.

### 1-D chaotic systems

Chaotic systems have applications in various engineering disciplines. They are used in secure communications, image and signal processing, and cryptography. One key aspect of chaotic encryption involves the use of Chaotic Maps. One-dimensional chaotic maps are particularly advantageous due to their simplicity, as they are governed by a single parameter, facilitating their implementation. Examples include logistics, sin, tent maps, and mathematical models exhibiting chaotic behavior. These maps generate pseudo-random sequences of values based on the iteration of simple mathematical equations. Equation (12) gives the general mathematical model of chaotic mapping^12^.

12 $${y}_{m+1}=f \left({y}_{m}\right)$$

here, $f \left({y}_{m}\right)$ represents a function with respect to ${y}_{m}$ where ${y}_{m}$ is the current state and ${y}_{m+1}$ denotes the next state*.* The initial state of the map is denoted as ${y}_{0}$, while the sequence of output values is given by {${y}_{1}$, ${y}_{2}$, ${y}_{3}$…}. In the context of discrete maps, the Lyapunov Exponent (LE) is defined as in Eq. (13).

13 $$\lambda = \underset{n\to \infty }{\text{lim}}\frac{1}{n} \sum_{i=0}^{n-1}\text{ln}\left|f{\prime}\left({x}_{i}\right)\right|$$

here, $f{\prime}\left({x}_{i}\right)$ represents the derivative of *f* (${x}_{i}$). If λ > 0, it indicates the presence of chaotic behavior in the system.

To improve the chaos complexity of existing 1-D chaotic maps and attain robust chaos,^18^ introduces a Sine Chaotification Model (SCM). Applying a sine function as a nonlinear chaotification transforms the outputs of a 1-D chaotic map. SCM improves the chaos complexity of the original chaotic map in the chaotic range and can cause chaos in the non-chaotic range.

1-D chaotic maps have been widely applied in cryptographic applications, such as image encryption^59–61^, key generation^62^ and general cryptographic systems^63,64^. Some studies have used chaotic systems in the design of S-Boxes^12,14,16,37,65^. The surveyed results indicate that 1-D chaotic maps are well-suited for S-Box generation due to their extensive range and excellent randomness characteristics. We avoid selecting multi-dimensional chaotic maps due to their increased complexity and the higher time costs associated with implementation, as they involve additional variables.

## Proposed S-boxes construction methodology

In the following subsections, a study of the different chaotic maps used as input functions in the proposed approach and an approach for S-box construction.

### Proposed chaotic map utilized in the proposed approach

**Fig. 1:**
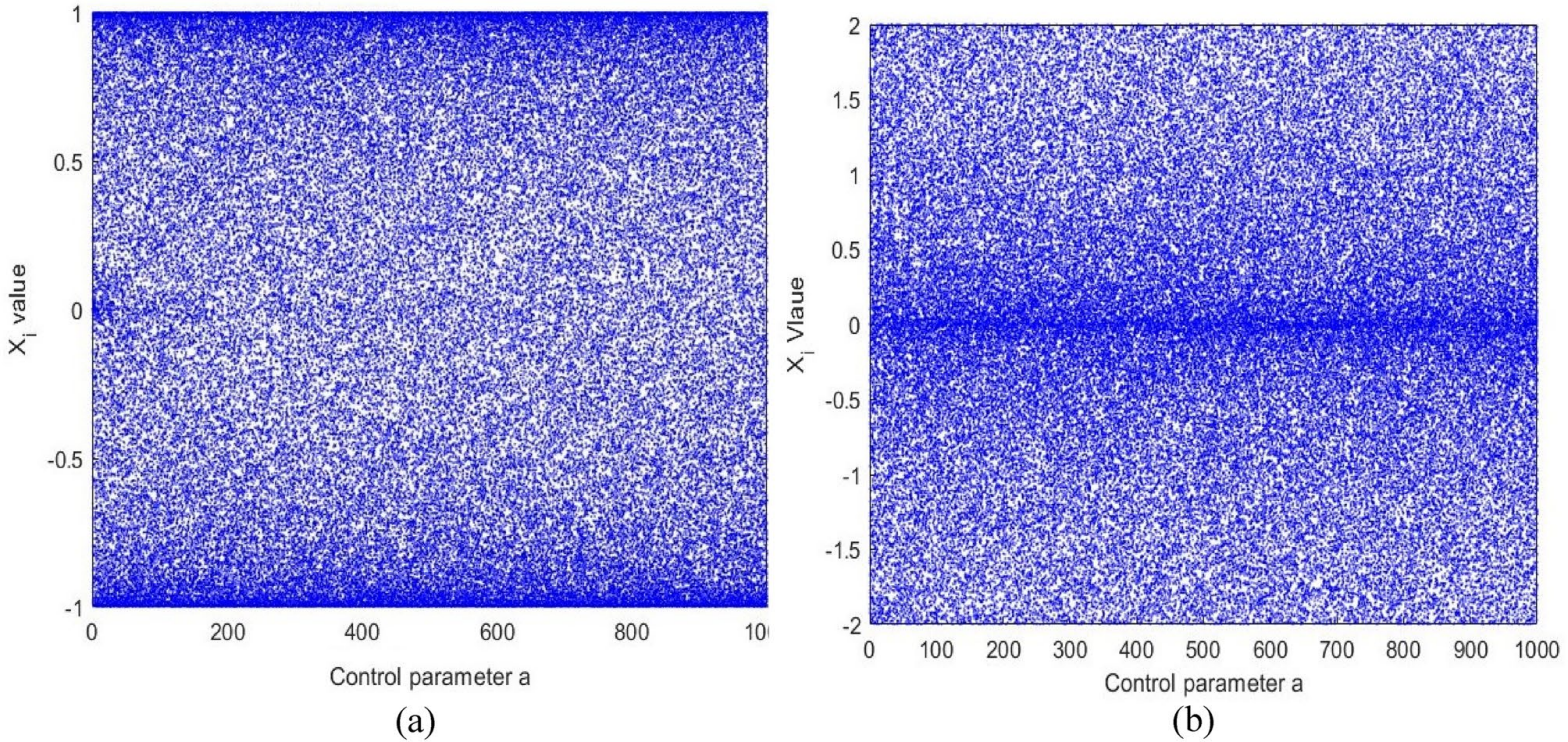
Bifurcation diagrams of the (**a**) ES map and (**b**) ELET map.

**Fig. 2:**
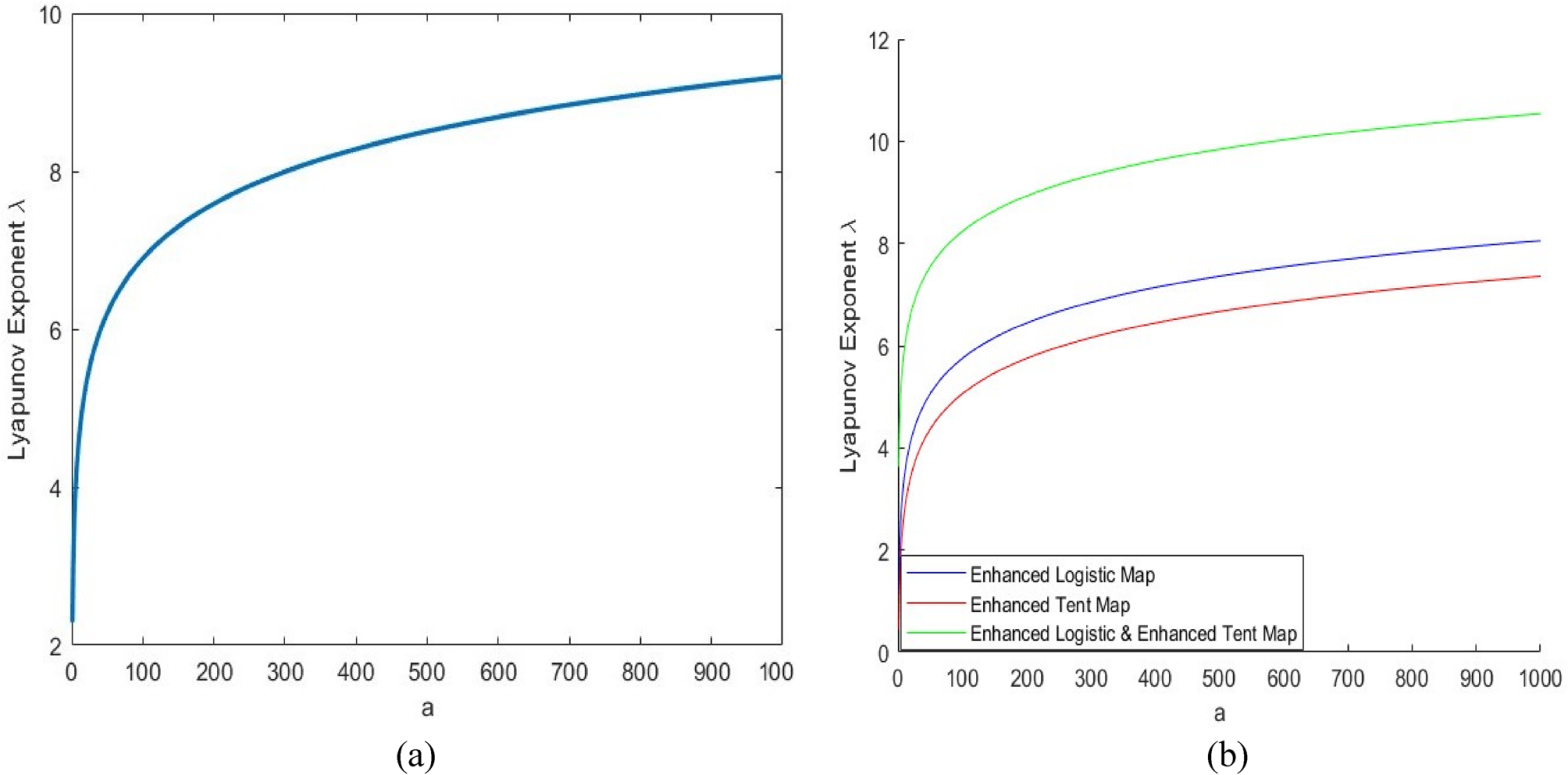
The Lyapunov Exponent LE of (**a**) ES map and (**b**) ELET map, along with comparisons with $\uplambda >0$ indicating chaotic behavior.

The research results in^37^ will be incorporated into our S-Box creation approach in this subsection. Initially, we conducted experiments with enhanced seed chaotic maps (Enhanced Logistic, Enhanced Tent and Enhanced Sin), and then we selected the best-performing one (Enhanced Sin, ES) defined as equation (14)^18^.

14 $${x}_{i+1}=\text{sin}\left(a\pi \text{sin}\left(\pi {x}_{i}\right)\right)$$

where *a* is the control parameter, and $\widetilde{\mu }\in \left(0,+\infty \right)$. Figure 1a plots the bifurcation diagram of the Enhanced Sin map when $\widetilde{\mu }\in \left[\text{0,1000}\right]$ and its output is randomly distributed within the data range of (-1,1). Using Eq. (15), the LE of the enhanced sin map is calculated. Figure 2a plots the LE of the enhanced sin map.

15 $$\lambda = \underset{n\to \infty }{\text{lim}}\frac{1}{n} \sum_{i=0}^{n-1}\text{ln}\left|\text{cos}\left(a\pi \text{sin}\left(\pi {x}_{i}\right)\right)a\pi \text{cos}\left(\pi {x}_{i}\right)\pi \right|$$

Subsequently, we combined the other maps, Enhanced Logistic (defined by equations (16)) and Enhanced Tent (defined by equation (17))^18^, resulting in a combined function (ELET), which is defined by equation (18). This function will be evaluated using metrics such as the Lyapunov Exponent (LE), bifurcation diagrams, and fixed points, ensuring that it continues to meet the criteria for chaotic behavior.

16 $${x}_{i+1}= \text{sin}\left(a\pi {x}_{i}\left(1-{x}_{i}\right)\right)$$

where *a* is the control parameter, and $\widetilde{a }\in \left(0,+\infty \right)$.

17 $${x}_{i+1}=\left\{ \begin{array}{c}\text{sin}\left(a\pi {x}_{i}\right), if {x}_{i}<0.5\\ \text{sin}\left(a\pi \left(1-{x}_{i}\right)\right), if {x}_{i}\ge 0.5\end{array}\right.$$

where *a* is the control parameter, and $\widetilde{r }\in \left(0,+\infty \right)$.

18 $${x}_{i+1}=\left\{ \begin{array}{c}\text{ssin}\left(a\pi {x}_{i}\right)+ \text{sin}\left(a\pi {x}_{i}\left(1-{x}_{i}\right)\right) , if {x}_{i}<0.5\\ \text{sin}\left(a\pi \left(1-{x}_{i}\right)\right)+ \text{sin}\left(a\pi {x}_{i}\left(1-{x}_{i}\right)\right), if {x}_{i}\ge 0.5\end{array}\right.$$

here, $a \in \left(0 , +\infty \right)$ is the control parameter, and ${x}_{i}$ lies within the range [− 2, 2].

Figure 1b presents the bifurcation diagram of the ELET function, which demonstrates a remarkably wide range of chaotic behavior, as observed. The LE of the ELET chaotic function is computed using Eq. (19).

19 $$\lambda = \underset{n\to \infty }{\text{lim}}\frac{1}{n} \sum_{i=0}^{n-1}\text{ln}\left| \left\{\begin{array}{c}a\pi \text{cos}\left(a\pi {x}_{i}\right)+a\pi \left(1-2{x}_{i}\text{cos}\left(a\pi {x}_{i}\left(1-{x}_{i}\right)\right)\right), {x}_{i}<0.5\\ -a\pi \text{cos}\left(a\pi \left(1-{x}_{i}\right)\right)+a\pi \left(1-2{x}_{i}\right)\text{cos}\left(a\pi {x}_{i}\left(1-{x}_{i}\right)\right), {x}_{i}\ge 0.5\end{array}\right.\right|$$

**Fig. 3:**
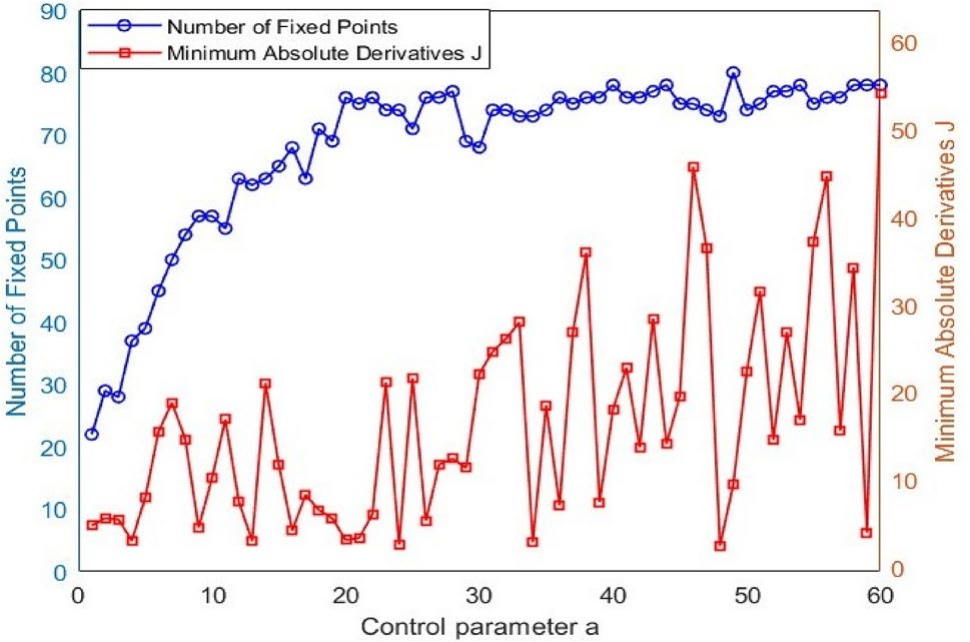
Number of fixed points with their minimum absolute derivatives of ELET map.

Figure 2b illustrates the LE of the ELET function. It shows that $\lambda >0$ against all values of the control parameter *a*, indicating that the given function exhibits chaotic behavior according to theory. By solving $f\left(x\right)=x$, we can determine the fixed-point values of the function described in Eq. (18). The multiple fixed points will be for *a* = 1 and *a* = 2. These fixed-point values can then be substituted into Eq. (20) to compute the corresponding derivative values, as shown in Table 1. The computation remains the same for $a>2$. Figure 3 illustrates the number of fixed points and the minimum absolute derivative for values ranging from 1 to 60. When $a\ge 1$, the number of fixed points increases, and their minimum absolute derivatives fall outside the range [-2, 2]. This implies that all fixed points of the ELET map are unstable. In a dynamic system, chaos emerges when all fixed points are unstable. Although concerns have been raised regarding the degradation of chaotic properties under finite-precision binary computations, this issue is effectively mitigated in the proposed algorithm. A combination of enhanced chaotic maps increases the system’s overall dynamic complexity and unpredictability. Furthermore, the control parameter is adaptively updated at each iteration, ensuring that chaotic behavior is preserved, and randomness is not degraded.

20 $$J= \frac{d{x}_{i+1}}{d{x}_{i}}=\left\{\begin{array}{c}a\pi \text{cos}\left(a\pi {x}_{i}\right)+a\pi \left(1-2{x}_{i}\text{cos}\left(a\pi {x}_{i}\left(1-{x}_{i}\right)\right)\right), {x}_{i}<0.5\\ -a\pi \text{cos}\left(a\pi \left(1-{x}_{i}\right)\right)+a\pi \left(1-2{x}_{i}\right)\text{cos}\left(a\pi {x}_{i}\left(1-{x}_{i}\right)\right), {x}_{i}\ge 0.5\end{array}\right.$$

**Table 1:** Fixed points along with associated derivatives of the ELET map.

Control element (*a*)	Fixed point (${x}_{i}$)	Associated derivative ($J$)
1	− 0.6516	− 8.4698
	0	6.2832
	0.8587	− 4.9296
2	− 1.3980	− 19.3443
	− 1.3219	18.0098
	− 1.1401	− 15.1495
	− 1.0454	18.5627
	− 0.3892	− 15.6319
	0	12.5664
	0.5438	6.0398
	0.5706	5.6480
	0.9236	− 10.3816
	1.6551	− 8.9976
	1.818	13.9040

This subsection briefly evaluates the proposed function’s chaotic properties to ensure its suitability for S−Box generation experiments; we do not focus on analyzing the chaotic function’s other aspects or comparing it to other functions. We aim to use chaotic maps with a wide range for experimenting in producing S-Boxes.

#### Proposed approach

**Fig. 4:**
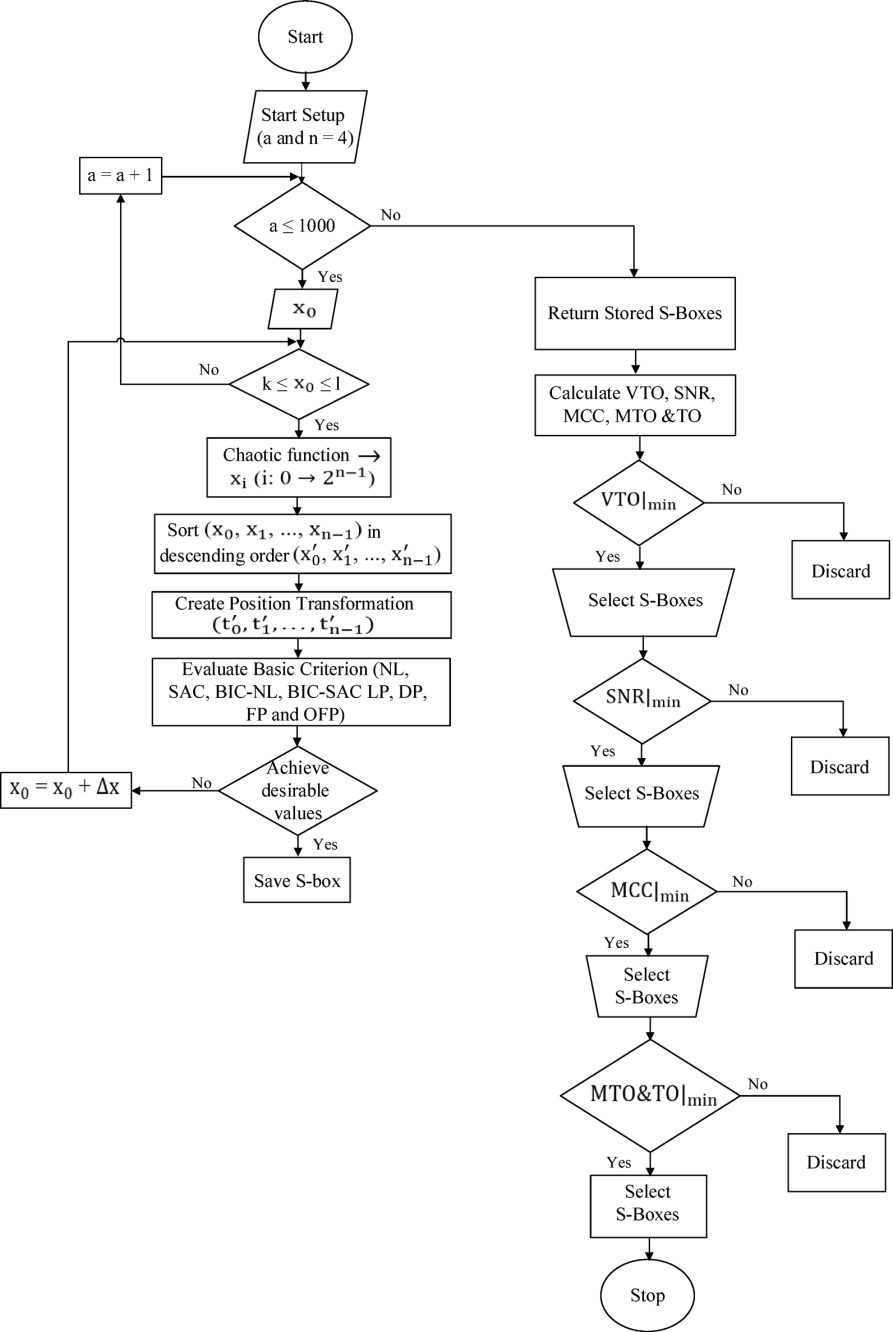
S-Boxes construction approach.

The approach for constructing S-Boxes with chaotic maps is presented in this subsection and shown in Fig. 4, and its corresponding algorithm is in Algorithm 1. The input function is derived from the enhanced seed functions in equations (14), (16), and (17), with equation (14) considered the best for constructing the S-Box that satisfies the desirable criterion, and therefore, it was selected. The combined function from Eq. (18) is then applied. Finally, the values and conditions employed during the initial phase of S-Box selection for various chaotic maps are explicitly provided in Table 2.

**Algorithm 1:**
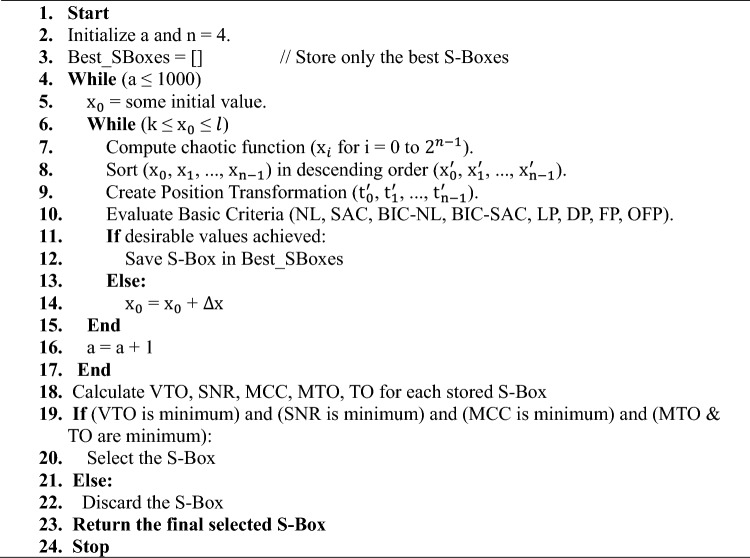
S-Boxes construction through proposed approach.

**Table 2:** The experimental parameters for different chaotic maps are applied in the proposed approach.

System-name	Equation number	a	${\mathbf{x}}_{0}$[$\mathbf{k},\mathbf{l}$]	$\Delta \mathbf{x}$
Enhanced Logistic map	[16]	[2,1000]; a = 2	[− 1,1];${\text{x}}_{0}$= − 1	0.0001
Enhanced Sin map	[14]	[2,1000]; a = 2	[− 1,1];${\text{x}}_{0}$= − 1	0.0001
Enhanced Tent map	[17]	[2,1000]; a = 2	[− 1,1];$\text{x}$= − 1	0.0001
ELET map	[18]	[1,1000]; a = 1	[− 2,2];${\text{x}}_{0}$= − 2	0.0001

The approach is split into two phases. The first phase optimizes the S-Boxes based on the main desired criterion, while the second phase optimizes SCA parameters to prevent adjacent channel attacks. The first phase aims to find a set of S-Boxes that optimizes the main parameters, including SAC, BIC-SAC, NL, DP, LP, FP and OFP, while satisfying the “Achieve desirable values” condition. The configurations of these parameters are based on standardized S-Boxes^37^ and are illustrated in Table 3. The algorithm ensures that S-Boxes are created and assessed in each iteration using different values of the control parameter *a*. If no valid S-Boxes are formed during the initial or subsequent iterations, the algorithm continues by updating the value of *a* until it reaches the maximum iteration count (1000). If no satisfactory S-Boxes are found throughout the process, the algorithm terminates without selecting any. Upon completing the first phase of the approach, we obtain a set of S-Boxes that satisfy the predefined criterion. In phase two, the generated set of S-Boxes is analyzed to calculate the SCA parameters, including VTO, MTO, TO, SNR, and MCC. Lower values of these parameters are preferred; therefore, we select the S-Box that satisfies the weakest values of VTO, SNR, MCC, MTO, and TO in this order, as derived from the results of several studies^45,58^. Nevertheless, thoroughly assessing the importance of each factor remains dependent on the specific attack scenario.

**Table 3:** Desirable values^37^.

*NL*	SAC	BIC-SAC	LP&DP	FP	OFP
$\ge 4$	= 0.5	= 0.5	$\le 0.25$	*0*	*0*

This approach has been implemented using the MATLAB programming language. To obtain the desired results during approach execution, the first chaotic function (ES), shown in Eq. (14), is configured with the parameters* a* = 597 and ${x}_{\text{o}}\approx -0.6763,$ while the proposed function, (ELET) shown in Eq. (18), is configured with *a* = 141 and ${x}_{\text{o}}\approx 1.6359$. Applying the proposed approach with these selected parameters generates two 4 × 4 S-Boxes using ES and ELET functions, as illustrated in Tables 4 and 5.

**Table 4:** Selected S-Box using the ES map.

x	0	1	2	3	4	5	6	7
S(x)	10	3	8	1	14	11	7	6
x	8	9	10	11	12	13	14	15
S(x)	4	12	2	0	5	15	13	9

**Table 5:** Selected S-Box using the ELET map.

x	0	1	2	3	4	5	6	7
S(x)	1	9	15	13	14	11	10	5
*x*	8	9	10	11	12	13	14	15
S(x)	6	12	4	0	2	8	3	7

### S-boxes security metrics

In this study, a MATLAB-based program was developed to analyze the key security metrics of S-Boxes and the parameters related to side-channel attacks. The program’s accuracy was verified by testing it on published S-Boxes such as PRESENT^22^ and FEATHER^41^. Following the criteria outlined in Section "Cryptographic properties of a strong S-box", this section analyzes the results related to the selected S-Boxes in Section "S-boxes security metrics". The parameters used for the tests are incorporated into the approach for S-Box generation. A thorough analysis and calculation of these parameters will be carried out after the S-Box selection process. A comparative analysis is conducted between our S-Boxes and alternative ones for each criterion. Since the parameters of the S-Boxes are dimensionless, all calculations and comparisons in our analysis are carried out without using units.

1. Balancedness: The truth tables of the Boolean functions for the two selected S-Boxes, Tables 4 and 5, contain an equal number of ones and zeros, indicating that the selected S-Boxes satisfy the balancedness property.
2. Bijective: Based on Eq. (1), the Boolean functions ${f}_{i}$ of the two selected S-Boxes satisfy the balancedness property, as they contain an equal number of zeros and ones. Furthermore, the S-Boxes produce all distinct output values from 0 to 15, thereby exhibiting the bijective property, which ensures that the selected S-Boxes are both injective and surjective.
3. Nonlinearity: To reduce the risk of linear cryptanalysis and preserve the confidentiality of plaintext, it is crucial to ensure a high degree of nonlinearity in the S-Box. Linear mappings between plaintext and ciphertext within an S-Box can make it vulnerable to such attacks. The nonlinearity of the Boolean functions that constitute the S-Boxes can be determined using Eqs. (2) and (3). Each of our 4 × 4 S-Boxes is composed of four Boolean functions. The results are summarized in Table 6. Furthermore, these Boolean functions can easily be converted into Algebraic Normal Form (ANF) to calculate the Algebraic Degree (AD) of the 4 × 4 S-Boxes, yielding an AD value of 3 for both selected S-Boxes. Thus, the constructed S-Boxes ensure resistance to algebraic analysis. However, some parameters related to the S-Box, such as Algebraic Immunity, Absolute Indicator, and Sum-of-Squares Indicator, are described in^41^, but we did not include them in this comparison table. The reason is that there is not much difference between the compared S-Boxes in terms of these parameters, making a comparative evaluation of these characteristics superfluous.

**Table 6:** Selected S-Boxes Boolean functions nonlinearities.

Boolean functions	${f}_{1}$	${f}_{2}$	${f}_{3}$	${f}_{4}$
ES S-Box NL	4	4	4	4
ELET S-Box NL	4	4	4	4
  Based on the presented data, the average nonlinearity (NL) of the selected S-Boxes is 4, representing the highest possible value. The selected S-Boxes achieve an NL value equivalent to that of the S-Boxes shown in Fig. 9, and attaining this value is straightforward.
4. Linear Approximation Probability: Using Eq. (6), we recalculated the LAP values for the two selected S-Boxes. For both S-Boxes, the most frequent occurrence of the input differential $\Delta x$ equaling the output differential $\Delta y$ was recorded four times in Fig. 5 and Fig. 6, which graph the Linear Approximation Table (LAT) of the selected S-Boxes. This yields an LAP of 0.25 for both S-Boxes.

**Fig. 5:**
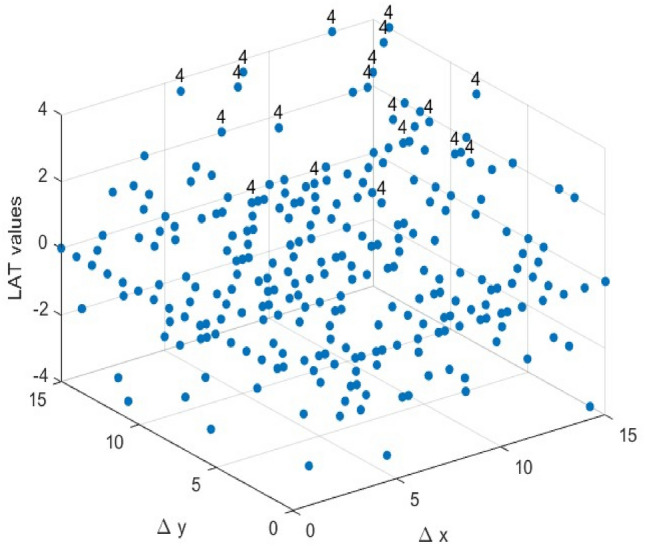
The LAT of the ES S-Box.

**Fig. 6:**
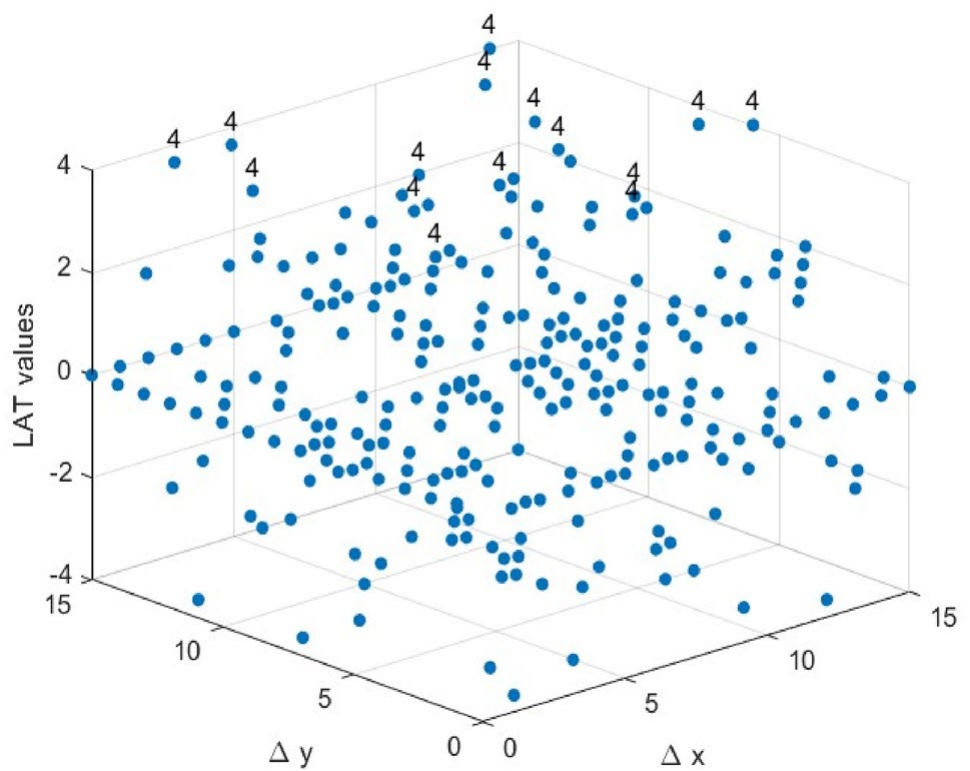
The LAT of the ELET S-Box.
  As shown in Fig. 9, all 4×4 S-Boxes share the same LP value, indicating that achieving an LP value of 0.25 is relatively straightforward for 4×4 S-Boxes. Therefore, while optimizing other parameters, the LAP value of the selected 4×4 S- Boxes remains competitive with other S-Boxes.
5. Differential Approximation Probability: The DDT can be used to calculate the S-Boxes’ DAP values. For a given input difference $\Delta x$, each entry in the table represents the frequency of the corresponding output difference $\Delta y$. Using Eq. (5), we will graph the DDT of the two selected S-Boxes, as shown in Figs. 7 and 8. We can calculate the DAP from the largest value in the DDT, which is 4/16 = 0.25. Based on the data in Fig. 9, the two selected S-boxes have the same DAP value as most other S-Boxes.

**Fig. 7:**
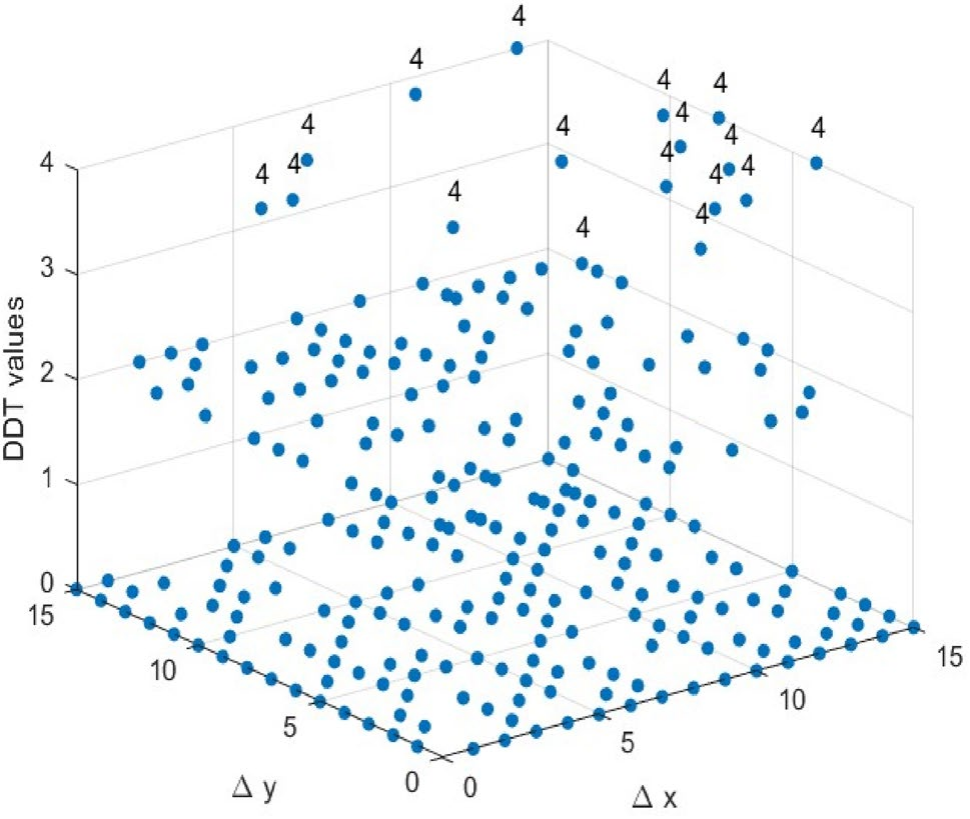
The DDT of the ES S-Box.

**Fig. 8:**
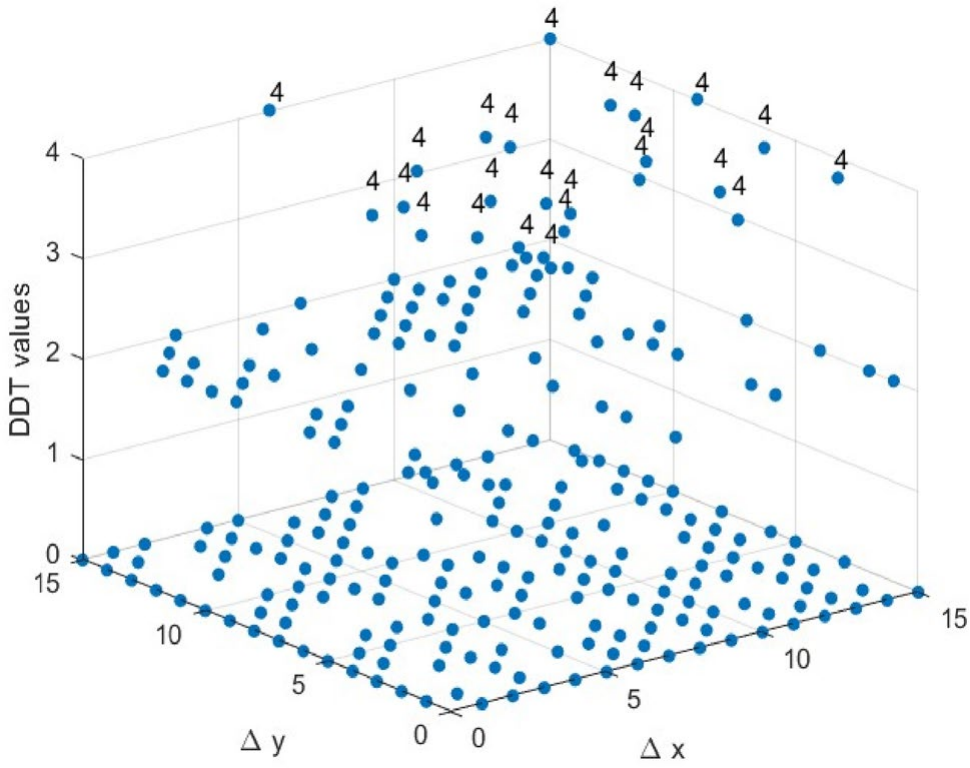
The DDT of the ELET S-Box.

**Fig. 9:**
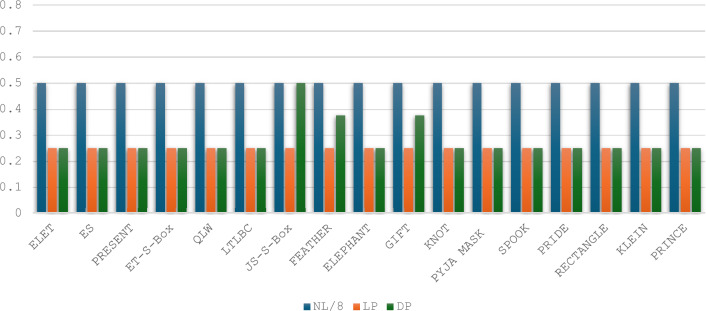
Comparison with other 4 × 4 S-Boxes in terms of NL, LP and DP.
6. Strict Avalanche Criterion: The SAC computations for the two selected S-Boxes were performed using Eq. (4), and the results are presented in Tables 7 and 8. Both S-Boxes were adjusted to meet the requirement of an average SAC value of 0.5, which is the optimal level. As shown in Fig. 10, the SAC comparison among various 4 × 4 S-Boxes reveals that three other S-Boxes achieved the optimal average SAC value of 0.5 in addition to the proposed S-Boxes: the ET-S-Box^37^, QLW^35^ and PRIDE^33^.

**Table 7:** Strict Avalanche Criterion (SAC) values for the ES S-Box.

i/j	1	2	3	4
1	0.50	0.25	0.75	0.50
2	0.25	0.50	0.50	0.75
3	0.25	0.50	0.50	0.50
4	0.50	0.50	0.50	0.75

**Table 8:** Strict Avalanche Criterion (SAC) values for the ELET S-Box.

i/j	1	2	3	4
1	0.25	0.25	0.50	0.75
2	0.50	0.50	0.50	0.50
3	0.50	0.75	0.75	0.50
4	0.50	0.50	0.25	0.50

**Fig. 10:**
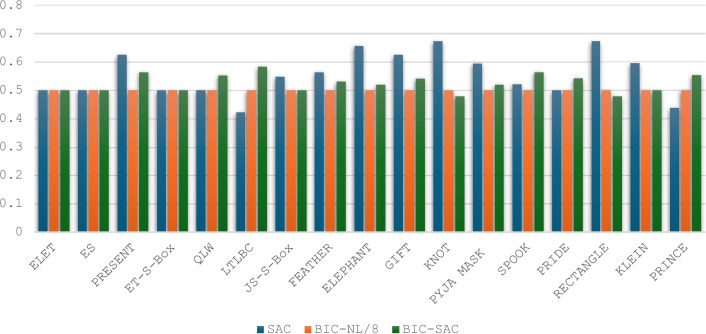
Comparison with other 4 × 4 S-Boxes in terms of SAC and BIC criteria.
7. Bit-Independent Criterion: The BIC-NL and BIC-SAC are advanced parameters used to evaluate the cryptographic strength of S-Boxes. Pairwise XOR operations are applied to all output functions to compute these values. The nonlinearity and SAC of these resulting functions are then calculated using the corresponding Eqs. (2), (3), and (4). The results are summarized in Tables 9, 10, and 11, showcasing the BIC-NL and BIC-SAC outcomes for the two selected S-Boxes. The analysis reveals that the functions for both S-Boxes achieve an optimal BIC-NL value of 4 and an optimal BIC-SAC value of 0.5. Consequently, applying the BIC criterion to the S-Boxes demonstrates optimal performance. As depicted in Fig. 10, the BIC-NL values are identical for the S-Boxes compared. Regarding BIC-SAC, three S-Boxes, along with the proposed S-Boxes, achieve the optimal value: ET-S-Box^37^, JS-S-Box^17^, and KLEIN^32^.

**Table 9:** Bit independent criterion results for nonlinearity (BIC-NL) for the two selected S-Boxes.

i/j	1	2	3	4
1	0	4	4	4
2	4	0	4	4
3	4	4	0	4
4	4	4	4	0

**Table 10:** Evaluation of BIC outcomes of SAC (BIC-SAC) for ES S-Box.

i/j	1	2	3	4
1	0	0.5625	0.5000	0.4375
2	0.5625	0	0.4375	0.5000
3	0.5000	0.4375	0	0.5625
4	0.4375	0.5000	0.5625	0

**Table 11:** Evaluation of BIC outcomes of SAC (BIC-SAC) for ELET S-Box.

i/j	1	2	3	4
1	0	0.4375	0.4375	0.5000
2	0.4375	0	0.6250	0.4375
3	0.4375	0.6250	0	0.5625
4	0.5000	0.4375	0.5625	0
8. Side-Channel Attack Resistance Metrics: The metrics related to the side-channel resistance of S-Boxes include VTO, SNR, MCC, MTO, and TO. Using Eqs. (7), (8), (9), and (11), we compute the TO, MTO, VTO, and SNR metrics for the two selected S-Boxes, as shown in Table 12. For the CC calculation, we apply Eq. (10), setting $${k}^{*}$$ = 0 during the computation. We then arrange ($${k}^{*},k)$$ in ascending order of magnitude. The results for the two selected S-Boxes are presented in Figs. 11 and 12. The MCC values for both S-Boxes are observed to be 0.125.

**Table 12:** S-Boxes side channel attack resistance metrics.

S-Box	TO	MTO	VTO	SNR	MCC
ES	3.4	1.8	1.867	1.664	0.125
ELET	3.6	1.533	1.867	1.612	0.125

**Fig. 11:**
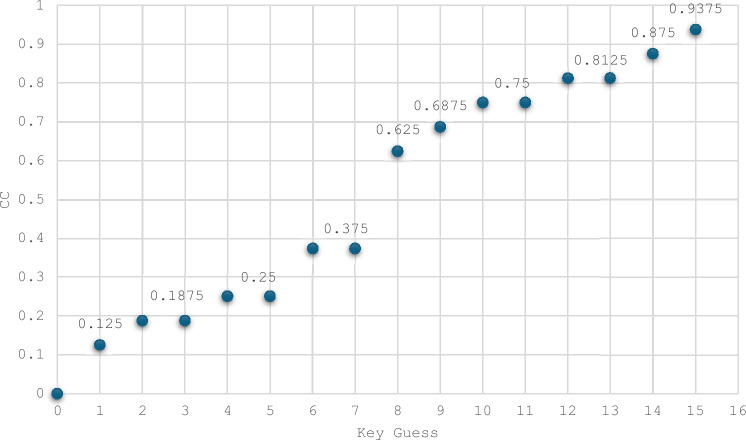
The confusion coefficient of the ES S-Box.

**Fig. 12:**
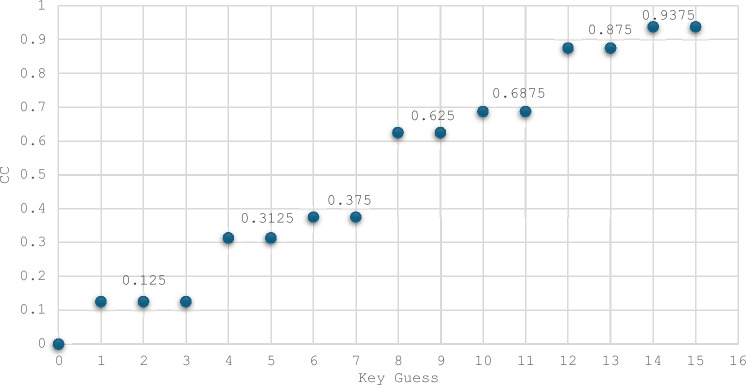
The confusion coefficient of the ELET S-Box.

**Fig. 13:**
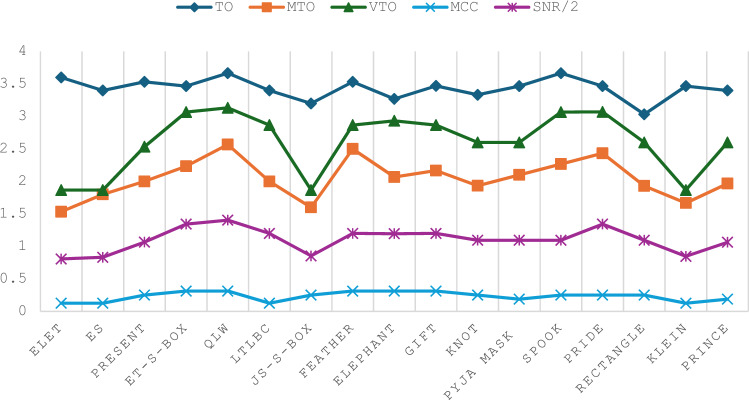
Side-channel attack resistance of 4 × 4 S-Boxes vs. the others.

Based on predefined values, the ELET S-Box outperforms the ES S-Box regarding MTO and SNR. While the ES S-Box shows an advantage in TO, this metric is considered less significant in the overall importance evaluation. Figure 13 compares 4×4 S-Boxes in terms of their resistance to side-channel attacks. Optimizing the VTO parameter is the primary focus of the proposed approach. The VTO results show that the proposed S-Boxes match the lowest value of 1.867, achieved by the JS-S-Box^17^ and the KLEIN S-Box^32^. Regarding the MTO values, it can be noticed that none of the S-Boxes achieve an MTO value as low as the proposed ELET S-Box. The ES S-Box only outperforms the JS-S-Box (1.6 compared to 1.8) and the KLEIN S-Box (1.667 compared to 1.8) in suppressing the MTO value. Regarding the TO values, the ES S-Box ranks fifth, with the smallest TO value among the other S-Boxes. Compared to MCC and SNR parameters, the proposed S-Boxes stand out with the lowest SNR value across all S-Boxes. Furthermore, the proposed S-Boxes achieve the lowest MCC value, along with the LTLBC and KLEIN S-Boxes. An overview of the comparison results is presented in Table 13. The proposed S-Boxes and the ET-S-Box achieve the ideal values of BIC-SAC (0.5) and SAC (0.5), as their designs focus on optimizing these specific criteria. However, while the ET-S-Box shows weak performance in resistance to side-channel attacks, the proposed S-Boxes excel in this area. Moreover, the proposed S-Boxes are designed to avoid Fixed Points and Opposite Fixed Points, enhancing their competitive edge compared to other S-Boxes based on various criteria. Parameters such as the Algebraic Degree were not included in the comparison table, as no significant differences were observed among the compared S-Boxes in this regard. The proposed S-Boxes have demonstrated exceptional performance across all evaluated criteria, surpassing others. Meeting essential benchmarks like NL, DP, and LP provides strong security against common block cipher attacks, including algebraic, linear, and differential attacks. The increasing focus on side-channel attacks in recent years highlights the need for S-Boxes that are resistant to such attacks. Therefore, this paper emphasizes optimizing key parameters during the S-Box design process to strengthen resistance against these threats. Our results show that the proposed approach for S-Box generation, whether using the enhanced sine map or combining the enhanced logistic and tent maps, is highly effective in producing robust and reliable S-Boxes. This method ensures strong security characteristics while remaining computationally efficient, though it is most suitable for S-Boxes of smaller dimensions due to the inherent randomness of the chaotic functions.

**Table 13:** Summarized comparison of the S-Boxes.

S-Box	Basic criteria						SCA Metrics					Ancillary	
	NL	LP	DP	SAC	BIC-NL	BIC-SAC	TO	MTO	VTO	MCC	SNR	FP	OFP
ELET	**4**	**0.25**	**0.250**	**0.500**	**4**	**0.500**	**3.600**	**1.533**	**1.867**	**0.1250**	**1.612**	**0**	**0**
ES	**4**	**0.25**	**0.250**	**0.500**	**4**	**0.500**	**3.400**	**1.800**	**1.867**	**0.1250**	**1.664**	**0**	**0**
PRESENT^22^	4	0.25	0.250	0.625	4	0.563	3.533	2	2.533	0.2500	2.130	0	1
ET-S-Box^37^	4	0.25	0.250	0.500	4	0.500	3.467	2.233	3.067	0.3125	2.726	0	0
QLW^35^	4	0.25	0.250	0.500	4	0.552	3.667	2.567	3.133	0.3125	2.808	0	2
LTLBC^36^	4	0.25	0.250	0.421	4	0.583	3.400	2	2.867	0.1250	2.398	0	2
JS-S-Box^17^	4	0.25	0.500	0.546	4	0.500	3.200	1.600	1.867	0.2500	1.706	0	2
FEATHER^41^	4	0.25	0.375	0.563	4	0.531	3.533	2.500	2.867	0.3125	2.398	1	0
ELEPHANT^26^	4	0.25	0.250	0.656	4	0.520	3.270	2.067	2.933	0.3125	2.390	0	1
GIFT^27^	4	0.25	0.375	0.625	4	0.541	3.470	2.167	2.867	0.3125	2.398	0	1
KNOT^28^	4	0.25	0.250	0.672	4	0.479	3.333	1.933	2.600	0.2500	2.188	0	2
PYJA MASK^29^	4	0.25	0.250	0.594	4	0.520	3.467	2.100	2.600	0.1875	2.188	0	2
SPOOK^30^	4	0.25	0.250	0.521	4	0.563	3.667	2.267	3.067	0.2500	2.188	1	2
PRIDE^33^	4	0.25	0.250	0.500	4	0.542	3.467	2.433	3.070	0.2500	2.188	1	2
RECTANGLE^34^	4	0.25	0.250	0.672	4	0.479	3.033	1.930	2.600	0.2500	2.188	0	0
KLEIN^32^	4	0.25	0.250	0.594	4	0.500	3.467	1.667	1.867	0.1250	1.692	0	0
PRINCE^31^	4	0.25	0.250	0.437	4	0.552	3.400	1.967	2.600	0.1875	2.128	0	1
Optimal value	High	Low	Low	0.500	High	0.500	Low	Low	Low	Low	Low	0	0

## Application and hardware efficiency

**Fig. 14:**
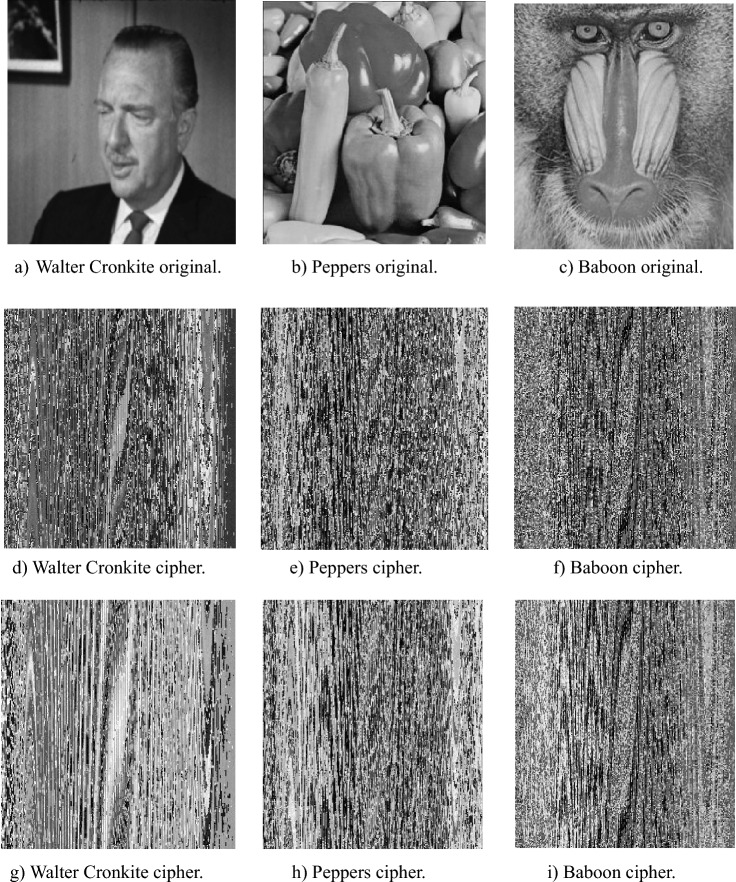
Non-encrypted images (**a**–**c**), encrypted images by ES S-Box (**d**–**f**) and encrypted images by ELET S-Box (**g**–**i**).

This section demonstrates the application of the proposed S-Boxes in image encryption, highlighting their effectiveness in securing image data. The process involves substituting pixel values of the image using the S-Boxes, followed by a pixel permutation to further obscure image features and eliminate recognizable patterns. The encryption is applied to three grayscale images, Walter Cronkite, peppers and Baboon, each sized 256 × 256, as shown in Fig. 14a–c. The input data consists of 256 × 256 8-bit grayscale values. Initially, each pixel’s 8-bit value is divided into two 4-bit nibbles. Pixel substitution is then performed on each nibble using the 4×4 S-Boxes, followed by a pixel permutation process. Finally, the resultant pixel values are used to reconstruct the encrypted image, as shown in Fig. 14d–i, demonstrating that the original image features are entirely concealed.

The effectiveness of encryption can be assessed through correlation analysis, which compares the pixels in the original and encrypted images. A lower correlation indicates a higher level of security, as it signifies that the encrypted image shares minimal similarity with the original. Equation (21) represents correlation mathematically^66,67^.

21 $$Correlation= \sum \left(\frac{\left(i- {\mu }_{i}\right)\left(j- {\mu }_{j}\right)}{{\sigma }_{i} {\sigma }_{j}}\right)$$

In addition to correlation analysis, we calculated the entropy of encrypted images as part of our expanded study. Entropy measures the randomness of data within an image. If the value

of the entropy is high, it means the data within the image is more disordered^68^. Equation (22) is utilized to measure the entropy^68^. The entropy value should be close to or equal to 8^69^.

22 $$Entropy= \sum_{i}P \left({\text{s}}_{i}\right) {\text{log}}_{2}\left(\frac{1}{P \left({\text{s}}_{i}\right)}\right)$$

where P (${\text{s}}_{\text{i}}$) represents the probability of pixel ${\text{s}}_{\text{i}}$ (i = 0 to 255) in an image.

Table 14 shows the correlation coefficients and entropy values for the three encrypted images. The correlation coefficients are all close to 0, indicating that the encrypted images exhibit minimal similarity to the original ones, which reflects strong encryption performance. Additionally, the entropy values are close to the ideal value of 8 for an 8-bit grayscale image, demonstrating a high level of randomness and confirming the effectiveness of the proposed S-Boxes in achieving strong confusion properties.

**Table 14:** Results of the correlation coefficients and entropy values.

Image	Average correlation while ES S-Box usage	Average correlation while ELET S-Box usage	Average entropy while ES S-Box usage	Average entropy while ELET S-Box usage
Walter Cronkite	− 0.044759	0.006661	7.662	7.591
Peppers	− 0.020057	− 0.004245	7.779	7.749
Baboon	− 0.012852	− 0.021633	7.584	7.500

The hardware efficiency of the ELET S-Box was then assessed by calculating its Gate Equivalent (GE). The process utilized the 45nm NanGate Open Cell Libraries (OCLs), which, as highlighted in^70^, provide an accurate benchmark for the area and complexity of logic gates implemented in 45nm CMOS technology. The ELET S-Box was first expressed as a set of Boolean functions to determine the GE. These functions were then optimized to achieve an efficient circuit design, minimizing the required logic gates and contributing to improved hardware efficiency. Table 15 gives an estimate of the Gate Equivalent (GE) of the logic gates employed in the aforesaid library, along with the implementation cost of our S-Box and its total latency.

**Table 15:** Area and Latency of logic gates in NanGate 45nm and ELET total cost.

Standard cell	Area (GEs)	Latency (ns)	ELET S-Box cost
NOT	0.67	0.022	5
AND2	1.33	0.040	7
AND3	1.67	0.051	3
OR2	1.33	0.056	3
OR3	1.67	0.085	2
XOR2	2	0.073	1
XNOR2	2	0.057	1
Total cost	29	1.011	–

To further analyze the S-Box’s power consumption characteristics, a complete simulation and evaluation methodology was used. The S-Box’s Register Transfer Level (RTL) description was written in Verilog and simulated with the ModelSim environment. A dedicated testbench was constructed to apply a variety of input vectors to the design, and the ensuing signal transitions during simulation were saved in a waveform file. This waveform data, which included the entire circuit’s switching activity, was then processed using a new Python-based analytic tool to estimate dynamic power consumption^71^. All supporting materials are prepared and ready for submission. Table 16 presents the proposed S-Box’s total power consumption compared to existing S-Boxes (PRESENT and RECTANGLE), demonstrating excellent energy efficiency with a total power of 5.076 μW while fully maintaining all functional requirements. The Gate Equivalent value, along with the associated latency and power consumption, is deemed appropriate for lightweight ciphers to achieve a balance between security and efficiency.

**Table 16:** Power analysis results.

S-Box	Total power(μW)
PRESENT S-Box^22^	4.320
RECTANGLE S-Box^34^	5.292
Proposed ELET S-Box	5.076

## Conclusion

A novel approach for constructing strong lightweight $4 \times 4$ S-Boxes essential for ensuring block ciphers’ security as their only nonlinear component has been presented. This study initially utilized the enhanced sin map, followed by a combination of the enhanced logistic map and enhanced tent map to generate an appropriate input for the S-Box construction approach. The proposed approach seamlessly incorporated multiple security criteria into the optimization process to generate strong S-Boxes, distinguishing it from other studies. Notably, this study successfully optimized both the BIC and SAC criteria for the S-Box, achieving a milestone not previously reached by any other S-Boxes. Additionally, the generated S-Boxes demonstrated excellent performance in meeting new criteria for resisting modern side-channel attacks while maintaining resilience against differential cryptanalysis, linear cryptanalysis, and algebraic attacks. Furthermore, they were also effective in image encryption, efficiently concealing image features. This study provided an in-depth analysis and evaluation of security criteria associated with S-Boxes, offering a more comprehensive approach than previous studies. With superior security properties, the proposed S-Boxes are suitable for integrating existing lightweight algorithms or developing new algorithms to secure embedded devices and IoT. Additionally, the proposed approach empowers developers to generate customized S-Boxes tailored to their specific security criteria requirements.

## Data Availability

The data used and/or analyzed during the current study are available from the corresponding author upon reasonable request.
